# Characterization of spontaneously generated prion-like conformers in cultured cells

**DOI:** 10.18632/aging.100370

**Published:** 2011-10-09

**Authors:** Roger S. Zou, Hisashi Fujioka, Jian-Ping Guo, Xiangzhu Xiao, Miyuki Shimoji, Crystal Kong, Cecilia Chen, Megan Tasnadi, Chesinta Voma, Jue Yuan, Mohammed Moudjou, Hubert Laude, Robert B. Petersen, Wen-Quan Zou

**Affiliations:** ^1^ Department of Pathology, Case Western Reserve University School of Medicine, 2085 Adelbert Road, Cleveland, Ohio 44106, USA; ^2^ Department of Pharmacology, Case Western Reserve University School of Medicine, 2085 Adelbert Road, Cleveland, Ohio 44106, USA; ^3^ Department of Neurology, Case Western Reserve University School of Medicine, 2085 Adelbert Road, Cleveland, Ohio 44106, USA; ^4^ Department of Neuroscience, Case Western Reserve University School of Medicine, 2085 Adelbert Road, Cleveland, Ohio 44106, USA; ^5^ Electron Microscopy Facility, Case Western Reserve University School of Medicine, 2085 Adelbert Road, Cleveland, Ohio 44106, USA; ^6^ Center for Mitochondrial Disease, Case Western Reserve University School of Medicine, 2085 Adelbert Road, Cleveland, Ohio 44106, USA; ^7^ National Prion Disease Pathology Surveillance Center, Case Western Reserve University School of Medicine, 2085 Adelbert Road, Cleveland, Ohio 44106, USA; ^8^ Kinsmen Laboratory of Neurological Research, University of British Columbia, Vancouver, BC V6T 1Z3, Canada; ^9^ Virologie Immunologie Moléculaires, UR892 INRA 78350 Jouy-en-Josas, France

**Keywords:** Prion protein, prion disease, insoluble prion protein, neuroblastoma cells, mutation, autophagy

## Abstract

A distinct conformational transition from the α-helix-rich cellular prion protein (PrP^C^) into its β-sheet-rich pathological isoform (PrP^Sc^) is the hallmark of prion diseases, a group of fatal transmissible encephalopathies that includes spontaneous and acquired forms. Recently, a PrP^Sc^-like intermediate form characterized by the formation of insoluble aggregates and protease-resistant PrP species termed insoluble PrP^C^ (iPrP^C^) has been identified in uninfected mammalian brains and cultured neuronal cells, providing new insights into the molecular mechanism(s) of these diseases. Here, we explore the molecular characteristics of the spontaneously formed iPrP^C^ in cultured neuroblastoma cells expressing wild-type or mutant human PrP linked to two familial prion diseases. We observed that although PrP mutation at either residue 183 from Thr to Ala (PrP^T183A^) or at residue 198 from Phe to Ser (PrP^F198S^) affects glycosylation at both N-linked glycosylation sites, the T183A mutation that results in intracellular retention significantly increased the formation of iPrP^C^. Moreover, while autophagy is increased in F198S cells, it was significantly decreased in T183A cells. Our results indicate that iPrP^C^ may be formed more readily in an intracellular compartment and that a significant increase in PrP^T183A^ aggregation may be attributable to the inhibition of autophagy.

## INTRODUCTION

The abnormal proteinase K (PK)-resistant prion protein (PrP^Sc^) is the only known component of the infectious prions that are associated with a group of fatal neurodegenerative disorders called prion diseases or transmissible spongiform encephalopathies [1]. While it has been well documented that PrP^Sc^ is derived from a PK-sensitive cellular PrP^C^ in the central nervous system through an alpha-helix to beta-sheet structural transition, the specific molecular mechanism(s) behind the PrP conversion remain poorly understood [2]. Several plausible theories have emerged, including the prevailing seeding model [3], which explains this conversion with the use of PrP^Sc^ seeds that are introduced either by exogenous infection in diseases such as kuru, iatrogenic Creutzfeldt-Jakob disease (CJD) and variant CJD, or formed by endogenous PrP^Sc^ molecules including sporadic CJD and various familial prion diseases. However, the exact molecular nature of the endogenous PrP^Sc^ has not been elucidated.

In 2006, we first identified small amounts of PK-resistant PrP aggregates in uninfected brains of humans, cattle and hamsters [4]. Since then, subsequent studies have revealed similar insoluble structures in a wide range of organisms, from cattle and sheep to humans [5] and cultured neuroblastoma cells expressing wild-type or mutant human PrP [6]. All these studies provide experimental evidence that prion-like forms are present at a low level in the normal brain. Such isoforms could be either the silent prions or prion precursors proposed previously [3, 7] or the recognized PrP species implicated in long-term memory in healthy humans [8]. Neuroblastoma cell expressing various human PrP mutants are an essential tool in the study of mutant prions linked to naturally occurring familial prion diseases [9-11, 6]. Previously, using two models expressing either the mutations at residue 183 from Thr to Ala (PrP^T183A^) or at residue 198 from Phe to Ser (PrP^F198S^), we discovered a PK-resistant PrP (PrP^res^) species that exhibited higher affinity for anti-PrP monoclonal antibody 1E4, with an epitope between residues 97 and 105 [6], compared to anti-PrP antibody 3F4, an antibody widely-used in the detection of human prions with an epitope between residues 106 and 112 [12]. Furthermore, we demonstrated that the immunoreactivity behavior and gel mobility of this PrP^res^ is virtually identical to those of PrP^*20^ that we observed previously in uninfected brain [4, 6], validating the cell model for the investigation of the spontaneously formed iPrP^C^.

In this study, using cells expressing PrP^Wt^, PrP^T183A^ and PrP^F198S^, we made the following observations. First, these PrP mutations increased aggregation of the protein, of which PrP^T183A^ that has been reported to accumulate intracellularly [10] exhibited the highest amount of iPrP. Second, immunofluorescence microscopy demonstrated that 1E4 had lower affinity for PrP in cultured cells than 3F4, as on immunoblots. Third, glycans at the second site (N197) are basic, whereas glycans at the first site (N181) are acidic. Fourth, the T183A mutation not only abolished glycosylation at N181, but also altered glycosylation at N197. In addition to the immaturity of the glycan at N197 reported previously [10], the composition of N197 may be also changed, as evidenced by the faster migration of N197 on PK-resistant PrP^T183A^ compared to that of N181 on the PK-resistant PrP^F198S^ or PrP^Sc^ from sCJD. Fifth, the F198S mutation also altered glycosylation at both the first and second sites. The glycosylation on the diglycosylated PrP^F198S^ seemed to be more mature than that on the monoglycosylated protein. Using monoclonal antibodies V14 and V61 that differentiate the two monoglycosylated PrP (Moudjou et al., 2004), we observed that the mutation blocked the V14 epitope, which was mimicked by reduction and alkylation, a reaction that often breaks disulfide bond and prevents re-oxidation of the thiol group [13]. Although V61 detected unglycosylated PrP and PrP monoglycosylated at N197 (mono197) in the brain PrP, it failed to detect the glycosylated and de- or un-glycosylated PrP from cultured neuronal cells. Finally, electron microscopy revealed that while cells expressing PrP^F198S^ enhanced autophagy, PrP^T183A^ seemed to completely inhibit autophagy compared to wild-type cells. Our findings provide new insights into the molecular mechanisms underlying the formation of iPrP^C^ and abnormal folding of mutant proteins.

## RESULTS

### Prediction of the effects of mutations on molecular weights, charges, and glycosylation of human PrP

We first predicated the effects of PrP^T183A^ and PrP^F198S^ on molecular weights and charges of the protein using the ProtParam tool at the website of http://web.expasy.org/protparam/ from the ExPASy; the molecular weight of the wild type prion protein (PrP^Wt^) backbone from residues 23 to 231 is 22,834.1 Dalton (Da). Compared to PrP^Wt^, the molecular weights of PrP^T183A^ and PrP^F198S^ were decreased (22,834.1 Da vs. 22,804.1 Da; 22,834.1 Da vs. 22,774.0 Da). Although the molecular weight of PrP^F198S^ decreased more than that of PrP^T183A^ compared to wild type controls, the small degree of change in the molecular weight might not be detectable by Western blotting. In addition, all three PrP molecules exhibited the same molecular charge with a theoretical isoelectric point (*p*I) of 9.39.

The N-linked glycosylation prediction algorithm NetNGlyc 1.0 was then used at http://www.cbs.dtu.dk/services/NetNGlyc to predict the likelihood of glycosylation at the two consensus sites. The NetNGlyc server provides a glycosylation potential value between 0 and 1 for each N-linked glycosylation site. A potential value greater than 0.5 indicates that glycosylation is probable at the site. Using this algorithm, the glycosylation potential values of N181 and N197 in PrP^Wt^ were determined to be 0.6636 and 0.7197, respectively. Notably, the potential value was greater at the second site than at the first site in all three species tested (PrP^Wt^, PrP^T183A^ and PrP^F198S^). Glycosylation at only N197, but not N181, was predicted in the PrP^T183A^ mutation (0.7196, almost identical to that of PrP^Wt^), while the first and second glycosylation sites were predicted for PrP^F198S^ (0.6633 and 0.6988, respectively). Interestingly though, the potential value at the second site was decreased for PrP^F198S^ compared to that in PrP^Wt^ (0.6988 vs. 0.7197), suggesting that the F198S mutation mainly affects glycosylation at N197. The expectation would be that the mutation from Phe to Ser at 198 would increase glycosylation [14].

### PK-resistant PrP conformers detected preferentially by the 1E4 anti-PrP antibody in cultured neuronal cells

As reported previously [10, 11, 6], PrP^T183A^ and PrP^F198S^ have altered gel profiles, largely because of the effect of the mutations on glycosylation. Probed with the 3F4 antibody directed against PrP106-110 [12], PrP^Wt^ migrated as three major bands of one di-glycosylated (~36-48 kDa), two mono-glycosylated (~34-36 kDa and ~32-33 kDa, respectively), and one un-glycosylated (27-28 kDa) forms (Fig. 1A, upper panel). In contrast, a single band migrating at 28-36 kDa, corresponding to the monoglycosylated PrP, was detectable in cell lysates from PrP^T183A^ expressing cells (Fig. 1A, upper panel). Bands migrating at 37-45 kDa and 33-36 kDa corresponding to the di- and mono-glycosylated forms, respectively, were detected in cell lysates from PrP^F198S^ expressing cells; the unglycosylated form was undetectable. From these data, we can conclude that PrP^T183A^ mainly contains monoglycosylated PrP at the second site (mono197), whereas PrP^F198S^ contains one di- and two separate mono-glycosylated PrP occupied at the first (mono181) or mono197. After treatment with PK at 25 μg/ml, no PrP was detected except from PrP^T183A^ expressing cells, in which a weak band with no change in migration remained when probed with 3F4. This band was un-digested PrP^T183A^.

**Figure 1:**
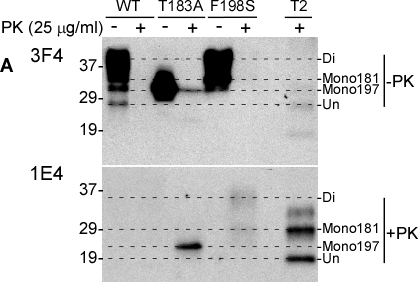
Detection of untreated and PK-treated PrP from three types of cultured cells with 3F4 and 1E4 ***A:*** Western blotting of cell lysates with or without PK-treatment at 25 μg/ml) probed with 3F4 (upper panel) and 1E4 (lower panel). WT: Lysates of cells expressing PrP^Wt^. T183A: Lysates of cells expressing PrP^T183A^ mutation. F198S: Lysates of cells expressing PrP^F198S^. T2: PrP^Sc^ type 2 control from sCJD. Di: Diglycosylated PrP. Mono181: PrP monoglycosylated at the first site. Mono197: PrP monoglycosylated at the second site. Un: Unglycosylated PrP. Comparison of affinities of 1E4 and 3F4 antibodies to the full-length and N-terminally truncated human PrP. The dashed-lines are used to align PrP bands on the blots.

In contrast, using the anti-PrP monoclonal antibody 1E4 directed against PrP97-105 [6], untreated PrP was virtually undetectable in all three cell lysates before PK treatment while 1E4 detected mutant, but not wild type, PK-resistant forms (Fig. 1A). One theory for this unique behavior of the 1E4 antibody is that the 1E4 epitope is blocked in full-length PrP, even when subjected to denaturing conditions prior to Western blotting, and becomes exposed only when truncated by PK with the removal of approximately 60-70 amino acids from the N-terminus [6].

In the cell lysates probed with 1E4, the profile of PK-resistant PrP^F198S^ was different from that of PrP^T183A^. In the T183A samples, 1E4 revealed an intense band migrating at 23-25 kDa corresponding to the PK-resistant monoglycosylated species (Fig. 1A, lower panel). PrP^F198S^ had two PK-resistant bands: a ~32-39 kDa band corresponding to di-glycosylated PrP and a ~26-29 kDa band corresponding to monoglycosylated PrP (Fig. 1A, lower panel). Interestingly, the mobility of di- but not mono-glycosylated PrP^F198S^ was slightly slower than that of PK-resistant PrP^Sc^ from sCJD (Fig. 1A, lower panel) while the mobility of the monoglycosylated PrP^T183A^ (second site) was faster not only than that of the monoglycosylated form of PrP^F198S^ from cell lysates, but also than that of PrP^res^ from the CJD brain control. Because the monoglycosylated PrP^T183A^ carries glycans at the second glycosylation site at residue 197, the monoglycosylated form of PrP^res^ from CJD brains and of PrP^F198S^ from cultured cells with slower migration may represent glycans at the first glycosylation site, residue 181. Another possibility is that the glycans at the N197 site are modified differently and consequently migrate slower than glycans from PrP^T183A^.

Utilizing immunofluorescence tagging and microscopy, we next compared cells expressing PrP^Wt^, PrP^T183A^ and PrP^F198S^ by immunostaining with 1E4 or 3F4. Consistent with Western blotting, all three cell types exhibited greater immunostaining with 3F4 than with 1E4 (Fig. 1B). Notably, although weak, PrP^Wt^ and two PrP mutants became detectable by immunofluorescence with 1E4, in contrast to results by Western blotting with 1E4 shown in Fig. 1A. In addition, as demonstrated previously, PrP^T183A^ is most likely located intracellularly, while wild type and PrP^F198S^ are present on the cell surface [10]. 3F4 immunostaining was reduced to almost insignificant levels when wild type cells were treated with PK (Fig. 1B), consistent with Western blotting results. In contrast, 1E4 immunostaining was decreased after PK-treatment, which was opposite to that observed in Western blots.

**Figure 1:**
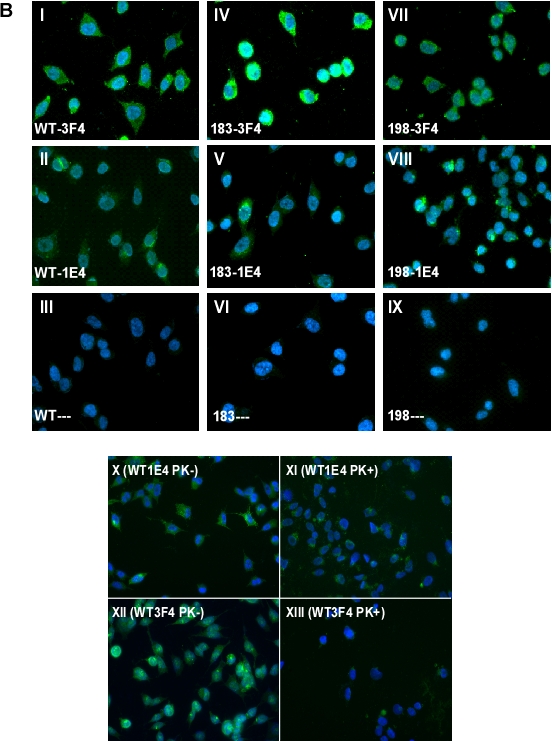
Detection of untreated and PK-treated PrP from three types of cultured cells with 3F4 and 1E4 ***B:*** Immunofluorescence detection of untreated and treated PrP with 3F4 and 1E4. Panels I-III: Cells expressing human PrP^Wt^. Panels IV-VI: Cells expressing human PrP^T183A^. Panels VII-IX: Cells expressing human PrP^F198S^. Panels I, IV, and VII: Staining with 3F4. Panels II, V, and VIII: Staining with 1E4. Panels III, VI, and IX: Staining without anti-PrP antibodies. Panels X and XI: Wild-type cells staining with 1E4 before and after PK-treatment. Panels XII and XIII: Wild-type cells staining with 3F4 before and after PK-treatment.

### Comparison of PrP oligomeric state between wild-type and mutant PrP

**Figure 2:**
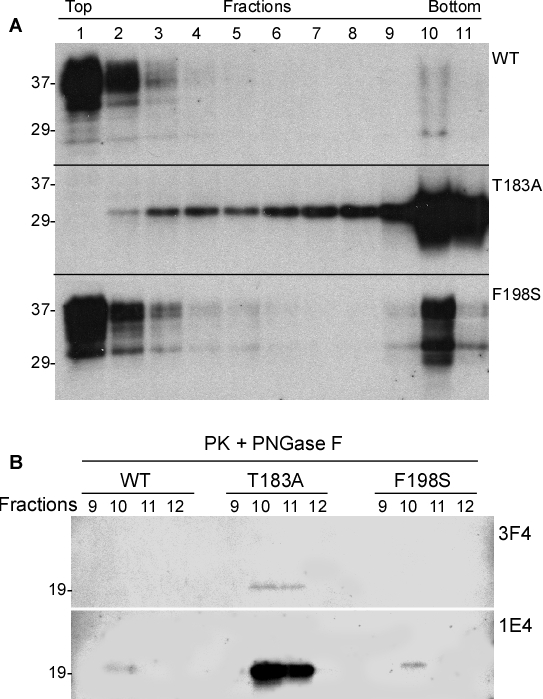
Detection of oligomeric state of wild-type and mutant PrP PrP from three types of cells were subjected to sucrose step gradient sedimentation. ***A:*** PrP in the fractions of the gradients was detected by Western blotting with 3F4. Upper panel: PrP^Wt^. Middle panel: PrP^T183A^. Lower panel: PrP^F198S^. ***B:*** PrP in fractions 9-12 was treated with PK and PNGase F prior to Western blotting with 3F4 (upper panel) and 1E4 (lower panel).

Sucrose step gradient sedimentation is a technique used to separate prion protein species based on their density, size, and conformation [15, 4]. In general, monomers or small oligomers are often recovered in the top fractions, whereas large aggregates are recovered in the bottom fractions after ultracentrifugation on the sucrose step gradients. An increase in the formation of PK-resistant PrP species in cells expressing mutant PrP might be associated with an increase in the aggregation of the protein. The distribution of PrP in 11 sucrose step gradients fractions collected after ultracentrifugation was determined by Western blotting probed with the 3F4 antibody. As expected, most PrP^Wt^ was recovered in the top fractions, i.e. fractions 1-2, whereas a small amount of PrP was observed in fraction 10. In contrast, significant amounts of PrP^T183A^ were recovered in the bottom fractions, whereas no PrP^T183A^ was detected in fraction 1. Only a very small amount of PrP was observed in fraction 2 (Fig. 2A, middle panel). Compared to PrP^Wt^, although most of PrP^F198S^ was distributed in the top fractions 1-2, an increased amount of PrP^F198S^ was also recovered in bottom fractions, especially in fraction 10 (Fig. 2A, lower panel).

To determine which fractions contain PK-resistant wild-type or mutant PrP, we treated PrP from fractions 1 to 11 with PK and PNGase F (Fig. 2B). PK-resistant PrP^T183A^ was only detected in fractions 10 and 11 when probed with 3F4; other fractions did not show any PK-resistance. In addition, 3F4 did not detect PK resistant wild type and F198S mutant PrP in any sucrose gradient fraction, while 1E4 detected PK-resistant iPrP in all three PrP species, with the highest intensity in the T183A mutant PrP (Fig. 2B). However, small amounts of PK-resistant PrP were only observed in fraction 10 for PrP^Wt^ and PrP^F198S^, whereas a larger amount of PK-resistant PrP was detected in fractions 10 and 11 for PrP^T183A^.

### Two-dimensional gel electrophoresis of iPrP^C^ from cultured neuronal cells expressing PrP^Wt^, PrP^T183A^, or PrP^F198S^

The above one-dimensional (1-D) electrophoresis and immunoblotting clearly indicated that glycans at the two N-linked glycosylation sites of the PK-resistant iPrP^C^ are different in their molecular weight: the monoglycosylated PrP species at the second site, residue 197 (mono197), migrated faster than those monoglycosylated at the first site, residue 181 (mono181). Taking advantage of the unique PrP^T183A^ mutation that eliminates the first but retains the second glycosylation site, we next asked whether mono197 and mono181 have distinct molecular charges by two-dimensional (2-D) gel electrophoresis, a technique that separates proteins based on both molecular weight and molecular charge. The gene 5 protein (g5p) was first used to isolate iPrP^C^ from the three types of cell lysates, following a previously described protocol [4, 6].

**Figure 3:**
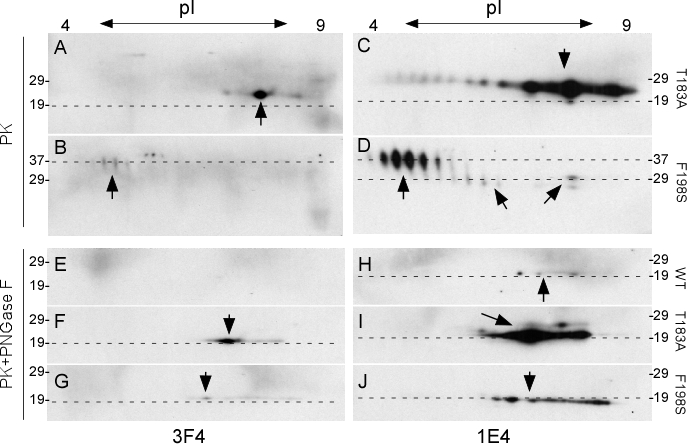
Two-dimensional gel electrophoresis and blotting of wild-type and mutant PrP captured by g5p and treated with PK or with PK plus PNGase F ***A*–*D:*** Cell lysates containing PrP^T183A^ (***A*** and ***C***) or PrP^F198S^ (***B*** and ***D***) were treated with PK at 25 μg/ml prior to Western blotting. ***E***-***J***: Cell lysates containing PrP^Wt^ (***E*** and ***H***), PrP^T183A^ (***F*** and ***I***), or PrP^F198S^ (***G*** and ***J***) were treated with PK plus PNGase F prior to Western blotting. PrP spots are highlighted by arrows. The dashed-lines are aligned with molecular weight makers. Blots ***A***, ***B***, ***E***, ***F***, and ***G*** were probed with 3F4, whereas blots ***C***, ***D***, ***H***, ***I***, and ***J*** were probed with 1E4.

Similar to 1-D immunoblotting, no convincing PK-resistant PrP^Wt^ spots were detected by 3F4 or 1E4 on 2-D blots (data not shown). However, the two PK-resistant mutants were detected by both 1E4 and 3F4, although the PrP intensity of the former was 5-10 fold greater using 1E4 compared to 3F4. The T183A mutant blots probed with 3F4 showed PK-resistant prion protein migrating at 23-26 kDa with isoelectric points (*p*I) from pH 8.0 to 9.5. An intense spot at pH 8.8 was also detected (Fig. 3A, arrow). These spots are believed to correspond to mono197 since the first glycosylation site is eliminated by the T183A mutation. The F198S mutant protein migrated at ~36-38 kDa with *p*I from pH 4.5 to 5.5. Based on their molecular weights, these spots represent di-glycosylated PrP species (Fig. 3B). However, spots detected using the 1E4 antibody were more intense and had noticeable differences on both blots. PrP^T183A^ spots detected by 1E4 migrating at ~22-27 kDa corresponding to mono197 were mainly detected in a range from pH 7.5 to 10.0 with faint spots also detectable at pH 4 (Fig. 3C). PrP^F198S^ spots were mainly detected between pH 4.5 and 6 migrating at ~35-39 kDa, corresponding to di-glycosylated forms (Fig. 3D). Two populations of faint PrP spots migrating at ~27-30 kDa were also detected between pH 6.2 and 7.2 as well as between pH 8.0 and 9.5, corresponding to mono181 or mono197. Spots migrating at ~27-30 could be PrP species monoglycosylated at the first site since they shared the similar molecular weight with mono181 identified by 1-D blotting (Fig. 1 and 3D), whereas spots between pH 8.0 and 9.5 migrating at ~24-26 kDa could be the monoglycosylated at the second site (Fig. 3D).

After treatment with PK and PNGase F, the two mutants exhibited PK-resistant forms, but not the wild type (Fig.3E-G). The PK-resistant PrP^T183A^ and PrP^F198S^ forms detected with 3F4 exhibited similar gel mobility at 19-20 kDa with pH between 7.0 and 9.5 on the blots, although there was a slight difference in pH between the most intense spots (Fig. 3F and 3G). However, in samples probed with the 1E4 antibody, the PK-resistant iPrP^C^ became detectable even for PrP^Wt^. The faint wild type spots migrating at ~19-20 kDa with pH 7.5-8.5 (Fig. 3H) while more intense PK-resistant PrP^T183A^ and PrP^F198S^ spots were detected with similar gel mobility migrating at ~19-20 kDa and pH 7-9.5 (Fig. 3I and 3J). Furthermore, these 2-D analyses of glycosylated and deglycosylated PK-resistant mutant iPrP indicated that glycans at the second site are more basic, whereas glycans at the first site are more acidic. 2-D blots, similar to the 1-D immunoblotting, also demonstrated that 1E4 has higher affinity for PK-resistant iPrP compared to 3F4. As indicated above, the effect of the two mutations by themselves on the charge of the proteins was not predicted. Two possibilities that may explain the slight differences in the pH of intense spots after deglycosylation: the three types of proteins have either distinct N-terminal protease cleavage sites or distinct GPI anchors.

### Detection of PrP^Wt^, PrP^T183A^, or PrP^F198S^ with anti-PrP antibodies directed against different N-linked glycosylation sites

Previous studies indicated that PK-resistant monoglycosylated PrP^T183A^ has a molecular weight lower than the PK-resistant monoglycosylated PrP^F198S^ or PrP^Sc^. This observation may be due to two possibilities: First, there may be abnormal glycosylation at N197 on PrP^T183A^, even though this mutation directly eliminates N181. Second, the molecular weight the glycans is greater in mono181 than in mono197. However, this hypothesis is inconsistent with a previous report [16]. In the previous study, two monoclonal antibodies termed V14 and V61 were generated using recombinant sheep PrP as an immunogen to produce glycan-controlled epitopes on the PrP molecule, which discriminate between the two monoglycosylated species [16, 17]. Notably, the epitopes recognized by the two antibodies were identified in 2.5-Å-resolution crystal structures of the PrP-antibody complex; the epitopes could not be mapped using conventional peptide scanning [16, 18, 17]. It was observed that the V14 epitope is localized between sheep PrP188 and 199 corresponding to human PrP185-196, whereas the V61 epitope has not been determined precisely yet. The V61 epitope was deduced to locate in the region before amino acid 171 to the first glycosylation site [16, 17]. Interestingly, although both antibodies recognize unglycosylated PrP, V14 binds to PrP with the first Asn glycosylation site occupied (corresponding to human mono181) and V61 binds to PrP carrying the second glycosylation site (corresponding to human mono197) from sheep, humans, hamsters, and mice. It was also reported that the glycan chain was smaller at the first glycosylation site than at the second site [16].

**Figure 4:**
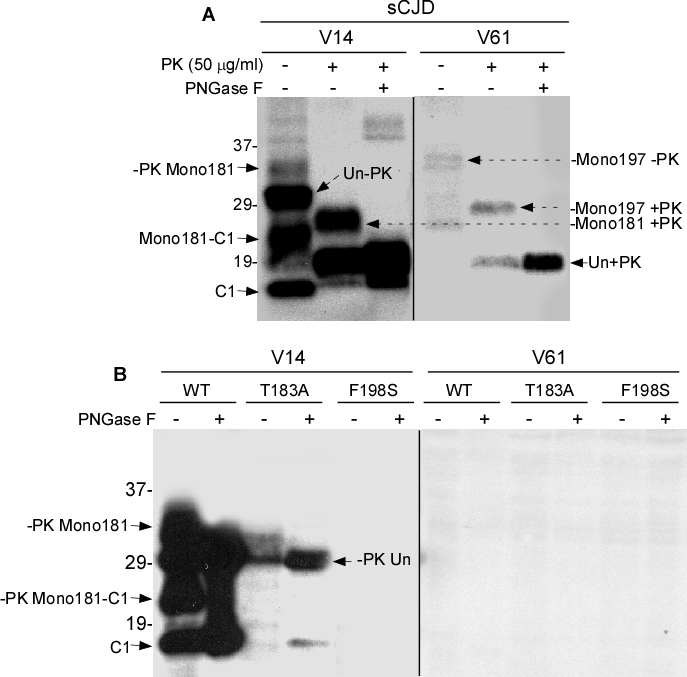
Western blotting of PrP with V14 and V61 ***A:*** PrP^Sc^ from sCJD untreated or treated with PK or PK plus PNGase F was probed with V14 or V61. ***B:*** PrP^Wt^, PrP^T183A^ or PrP^F198S^ from cells untreated or treated with PNGase F was probed with V14 or V61.

We first examined PrP^Sc^ that was untreated, treated with PK alone, or treated with PK plus PNGase F from sCJD brains with the two antibodies. Without PK or PNGase F treatment, five major PrP bands were detected by V14 (Fig. 4A). Based on the molecular weight, the top band migrating at ~33-35 kDa represents the monoglycosylated PrP species. Since V14 detects PrP species monoglycosylated at N181, this band is mono181. The second band migrating at ~29-32 kDa corresponds to the unglycosylated full-length PrP. The third band migrating at ~ 23-25 is the monoglycosylated C1 fragment with the first site occupied (mono181- C1). The fourth band migrating at 19-20 kDa is the unglycosylated C2 fragment, an endogenously N-terminally truncated PrP fragment detected in CJD brains [19]. Finally, the fifth band migrating at ~18 kDa is unglycosylated C1 that is N-terminally truncated in normal brains [19]. After PK-treatment, only three major bands are detectable with V14. The top band migrating at ~26-28 is PK-resistant PrP monoglycosylated at the first site (mono181 +PK). The second band migrating at ~19 kDa is PK-resistant unglycosylated PrP (Un + PK). The third faint band was detected at ~18.5 kDa. In the samples treated with both PK and PNGase F, only two bands were detectable: the dominant band migrated at ~19-20 kDa, while the other migrated at ~18.5 kDa. Compared to the samples treated with PK, but without PNGase F, the band migrating at ~18.5 kDa significantly increased, suggesting that this new fragment contains both glycosylated and unglycosylated forms. Moreover, since it was not detected in the untreated samples, this new fragment could be generated by PK digestion *in vitro* (Fig. 4A, left panel).

Although equal amounts of samples were used, in general, the PrP intensity was much lower in samples probed with V61 than in samples probed with V14 (Fig. 4A, right panel). The intensity of PrP bands in the untreated PrP detected with V61 was weak and four bands could be identified. The top double bands migrating at 34-36 kDa corresponds to the PrP species monoglycosylated at the second glycosylation site (mono197 -PK). The band migrating at ~29-31 kDa corresponds to the unglycosylated full-length PrP. The band migrating at ~27-28 kDa represents C1 monoglycosylated at the second site (mono197-C1). After treatment with PK, two bands became detectable migrating at ~29-30 kDa and 19-20 kDa, respectively. Since V61 detected the monoglycosylated species occupied at residue 197, they represent the PK-resistant PrP species monoglycosylated at the second site (mono197 +PK) and PK-resistant deglycosylated and unglycosylated forms (Un +PK). Consistent with the observation by Moudjou et al., the migration of the PrP mono197 detected with V61 was slower than that of PrP mono181 detected with V14 (Fig. 4A). Therefore, using the two antibodies, we observed that the human PK-resistant PrP species monoglycosylated at the second site (mono197) has a higher molecular weight than that monoglycosylated at the first site (mono181). In addition, both antibodies were unable to detect di-glycosylated human PrP, consistent with the previous observation by Moudjou et al. [16].

In cultured neuronal cell lysates containing PrP^Wt^, four major bands were detected by V14, migrating at ~33-35 kDa, ~29-32 kDa, ~23-25 kDa, and ~18 kDa (Fig. 4B, left panel). They correspond to mono181, unglycosylated PrP, mono181-C1, and unglycosylated C1. Compared to PrP^Sc^ from sCJD in Fig. 4A, C2 was dramatically decreased in uninfected cell lysates. After treatment with PNGase F, only two bands were detected, corresponding to deglycosylated full-length at ~29-32 kDa and N-terminally truncated C1 at 18 kDa. V14 mainly detected the unglycosylated PrP^T183A^ migrating at ~29-32 kDa. Faint bands were also detected migrating at ~19 kDa and 18 kDa corresponding to C2 and C1, respectively (Fig. 4B, left panel). After deglycosylation with PNGase F, two bands corresponding to deglycosylated and unglycosylated full-length and N-terminally truncated PrP molecules were detected by V14. In contrast, no bands were detected by V14 in treated and untreated cell lysates containing PrP^F198S^. Surprisingly, no PrP bands were detected by V61 in all three cell lysates untreated and treated with PNGase F (Fig. 4B, right panel). Using the two antibodies we were unable to address issues why the migration of the mono197 from PrP^T183A^ is faster than that of PrP^F198S^ because of inability of V14 to detect PrP^F198S^ and because of inability of V61 to detect both wild-type and mutant PrP from cultured cell lysates. Nevertheless, our current study revealed that V14 identified a C1 monoglycosylated at the second glycosylation site (mono197-C1). Moreover, our new results for the first time indicated that F198S mutation blocked the binding of V14 to PrP and the V61 epitope was completely blocked in both wild-type and mutant PrP from cultured cells.

### Effect of reduction and alkylation on the accessibility of the V14 epitope and epitope mapping with a peptide array

The inability of V14 to detect PrP^F198S^ raises two possibilities: First, the residue Phe itself is a key element of the epitope and the F198S mutation completely blocks V14 binding. However, the residue Phe is out of the V14 epitope, as demonstrated by the X-ray crystallography study [18, 17], which does not support this possibility. Second, although residue Phe itself is not part of the epitope, F198S mutation may affect the disulfide bond between residues 179 and 214, which blocks the accessibility of the V14 epitope. To rule out this possibility, we next used V14 to detect PrP treated with a reducing reagent tributylphosphine (TBP) and an alkylating reagent mechlorethamine (MCT) that break disulfide bond and block the free thiol group.

**Figure 5:**
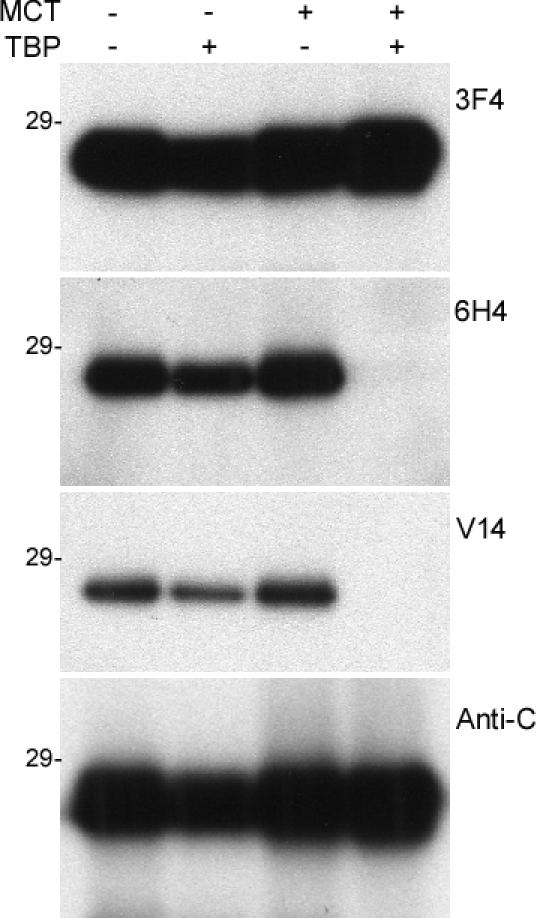
Western blotting of the recombinant human PrP untreated or treated with MCT or/and TBP probed with various anti-PrP antibodies The recombinant human PrP23-231 was treated with MCT alone, TBP alone, or MCT plus TBP prior to Western blotting with 3F4, 6H4, V14 and V61.

In addition to the V14 antibody, three other antibodies were used as controls: 3F4 directed against PrP105-112, 6H4 directed against PrP145-152 and anti-C against PrP220-231. All antibodies detected untreated recombinant human PrP23-231, while they also detected PrP in samples treated with TBP or MCT alone (Fig. 5). Treatment with TBP alone decreased the intensity of PrP probed with the four antibodies. However, V14 detected no PrP at all in samples treated with TBP and MCT together, while 3F4 and anti-C antibodies still detected PrP (Fig. 5). This result is similar to our previous observation with 6H4 [13, 20].

We previously used peptide membrane array, a technique that has often been used to map antibody epitopes, to re-map epitopes of 1E4 and 3F4 antibodies [6, 12]. However, here we were unable to find convincing epitopes for V14 and V61 antibodies using a 99 13-mer peptide membrane array that covers the entire human PrP sequence from residues 23 to 231 (data not shown). Our result is consistent with the observation reported by Moudjou et al. [16], suggesting that they have conformational epitopes even though they are able to detect the denatured PrP on Western blots.

### Comparison of glycan modification between PrP^T183A^ and PrP^F198S^ by treatment with Endo H and PNGase F

**Figure 6:**
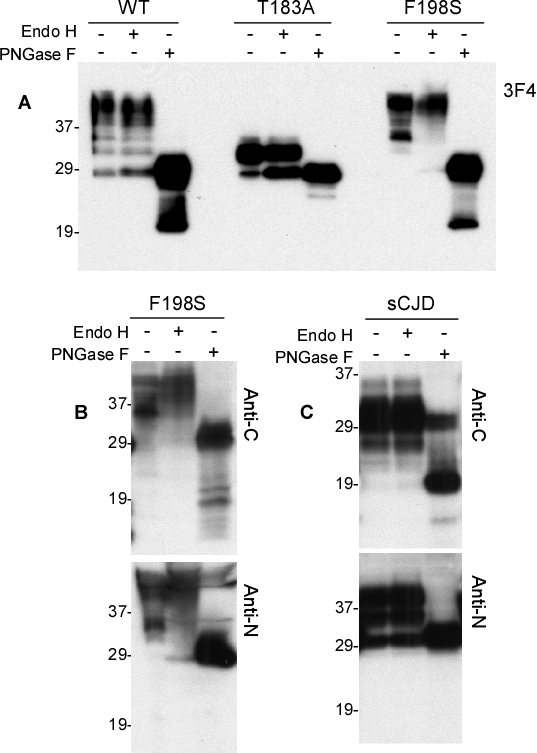
Digestion of wild-type and mutant PrP by Endo H ***A:*** PrP^Wt^, PrP^T183A^, or PrP^F198S^ was treated with Endo H or PNGase F prior to Western blotting with 3F4. ***B:*** PrP^F198S^ was treated with Endo H or PNGase F prior to Western blotting with Anti-C or anti-N. ***C:*** PrP^Sc^ from sCJD was treated with Endo H or PNGase F prior to Western blotting with anti-C or anti-N.

We treated PrP^Wt^, PrP^T183A^ and PrP^F198S^ with Endo H, or PNGase F that cleaves N-linked glycans. The Endo H treatment did not significantly change the gel profile and glycoform ratios of PrP^Wt^ (Fig. 6A). In contrast, it did significantly reduce the intensity of the monoglycosylated PrP^T183A^ and increase the intensity of its unglycosylated form (Fig. 6A). No relevant changes in the intensity of di-glycosylated PrP^F198S^ were observed, while the two monoglycosylated PrP^F198S^ species became undetectable after treatment with Endo H, suggesting that Endo H only removes glycans from monoglycosylated PrP at the two sites. On the other hand, a faint band migrating at ~28-29 kDa corresponding to the unglycosylated and deglycosylated PrP^F198S^ was visible in the treated sample, probably deriving from monoglycosylated PrP (Fig. 6A). After treatment with PNGase F, all three different PrP species were observed to have a band migrating at ~28-29 kDa corresponding to deglycosylated and unglycosylated PrP (Fig. 6A). However, only PrP^Wt^ and PrP^F198S^ exhibited a band migrating at ~20 kDa corresponding to endogenously N-terminally truncated fragment C2.

To confirm that Endo H only reduces monoglycosylated but not di-glycosylated PrP species, we proceeded to examine Endo H or PNGase F treated PrP^F198S^ with two more antibodies including anti-C and anti-N (Fig. 6B). Reaffirming evidence from the 3F4 antibody, both anti-C and anti-N antibodies also revealed no decreases in the intensity of the di-glycosylated PrP but dramatic decreases in the intensity of the monoglycosylated PrP species. PNGase F also removed all glycans from PrP^F198S^. Using these two antibodies, we also examined PrP^Sc^ from sCJD and observed no changes in the intensity of both di- and mono-glycosylated PrP species after treatment with Endo H (Fig. 6C). Once again, PNGase F removed glycans from PrP^Sc^ as well.

### Electron microscopy of three types of cultured cells

**Figure 7:**
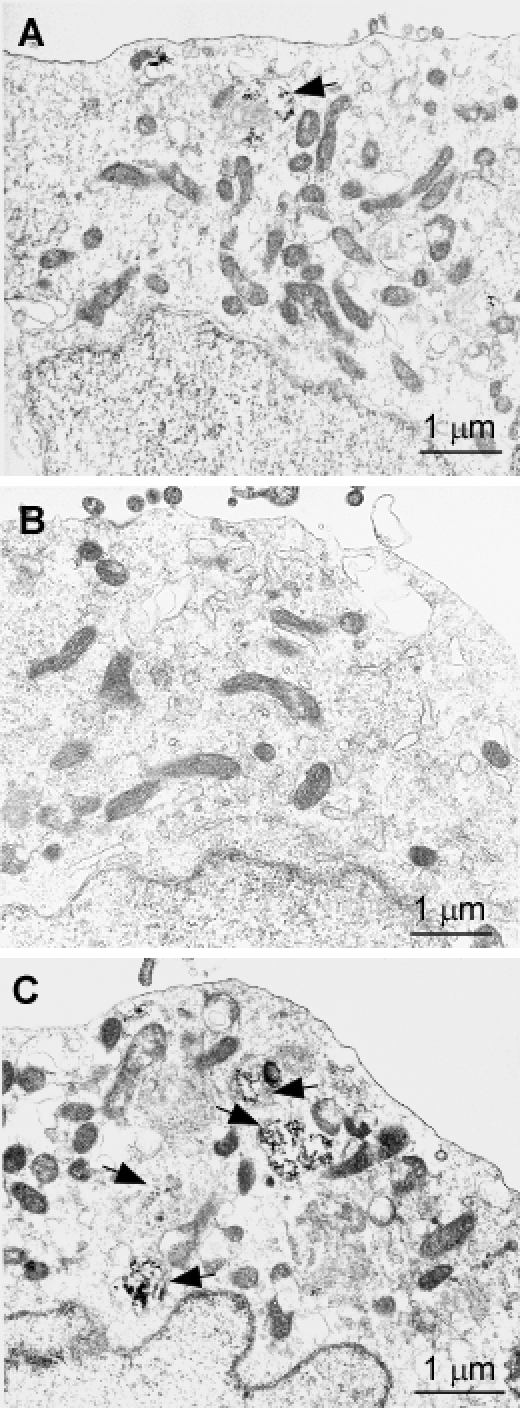
Transmission electron microscopy of cells expressing PrP^Wt^, PrP^T183A^, or PrP^F198S^ ***A:*** Cells expressing PrP^Wt^. ***B:*** Cells expressing PrP^T183A^. ***C:*** Cells expressing PrP^F198S^.

Since the two mutations significantly increased PrP aggregation, as evidenced above by protein chemistry studies, we asked whether there are any changes in organelles and cellular structures induced by mutant PrP that readily formed aggregates. Cells expressing PrP^Wt^, PrP^T183A^, or PrP^F198S^ were examined by electron microscopy (Fig. 7). Although most organelles including mitochondria and nucleus seemed to be identical between these cells, the lysosome had characteristic differences between cells expressing the various PrP species. Compared to PrP^Wt^ cells (Fig. 7A), PrP^F198S^ cells exhibited enhanced autophagy (Fig. 7C), while virtually no autophagy was observed in PrP^T183A^ cells (Fig. 7B).

## DISCUSSION

The two N-linked glycosylation sites located at residue 181, Asn-Ile-Thr residues 181-183, and residue 197, Asn-Phe-Thr residues 197-199 [21] have been believed to play a crucial role in the stabilization of prion protein conformation. The naturally occurring mutations at residue 183, Thr to Ala (PrP^T183A^), or at residue 198, Phe to Ser (PrP^F198S^), falling in the two consensus sites are linked to two distinct familial prion diseases [22, 23]. Elimination of each of the two sites or two sites together by mutagenesis of hamster PrP in CV1 cells induced the intracellular accumulation of mutant proteins [24]. Lehman and Harris observed that mouse PrP mutated at T182 alone or at both T182 and T198 in CHO cells failed to reach the cell surface but those mutated at T198 did. Moreover, all three mutant PrP acquired PrP^Sc^-like physicochemical properties reminiscent of PrP^Sc^ while to a limited extent PrP^Wt^ did so only when synthesized in the presence of inhibitor tunicamycin [25]. Using M17 cells expressing human PrP^N181G^ or PrP^T183A^, Capellari et al. observed that PrP^N181G^, but not PrP^T183A^, reached the cell surface even though both mutations eliminated glycosylation at the first site [10]. This indicates that the Thr to Ala mutation itself, not the elimination of the first glycosylation site, altered the physical properties of the mutant protein [10]. Although the F198S mutation falls within the second glycosylation site, Asn-Phe-Thr residues 197-199, PrP^F198S^ slightly increased the efficiency of glycosylation at the first glycosylation site (N181), and greatly increased the efficiency of glycosylation at the second site (N197) in cultured cells [11].

Our current study indicated that the two mutant PrP molecules exhibited increased aggregation, although a small amount of PrP^Wt^ aggregates is also evident in the absence of tunicamycin. Most of PrP^T183A^ was in oligomers and large aggregates; virtually no monomeric form was present. In PrP^F198S^, however, monomeric species were still dominant despite an increase in the amount of aggregates, since the majority of PrP^F198S^ was recovered in the top fractions of sucrose step gradients. The enhanced tendency of PrP^T183A^ to form aggregates may result from the intracellular accumulation of the mutant protein. The F198S mutation did not change the ability of PrP^F198S^ to reach the cell surface [11], although the mutation may significantly change the structure around the V14 epitope previously found to be localized between human PrP185-196 [16, 17]. Therefore, the majority of the iPrP^C^ associated with the T183A mutation may be the result of its PrP intracellular accumulation, raising the possibility that iPrP^C^ is derived predominantly from intracellular PrP species. Immunofluorescence microscopy of tagged PrP also indicated that PrP^T183A^ accumulates within the cell, whereas PrP^F198S^ was distributed both inside the cell and on the cell surface, consistent with previous observations [10, 11].

In addition to abnormal protein folding, the two mutations also significantly altered N-linked glycosylation. As anticipated by the N-linked glycosylation prediction algorithm NetNGlyc 1.0 and confirmed by Western blot analysis, PrP^T183A^ completely abolishes glycosylation at N181. However, PrP^T183A^ also exhibited altered glycosylation at N197. On 1-D blots, the migration of the PK-resistant mono197 from PrP^T183A^ was faster than that of PrP^F198S^ and of the dominant monoglycosylated PrP^Sc^ from sCJD, suggesting that the apparent molecular weight of glycans at mono197 is smaller than that of glycans at mono181. However, using two monoclonal antibodies V14 and V61, we demonstrated that the apparent molecular weight of PK-resistant mono197 of PrP^Sc^ from sCJD is greater than that of mono181 of PrP^Sc^, consistent with the previous finding in scrapie PrP^Sc^ [16]. Thus, the combination of these results provides further evidence to suggest the altered composition of glycans at N197 of PrP^T183A^. Furthermore, our 2-D analyses revealed that *p*Is of PK-resistant PrP^T183A^ are basic, while the PK-resistant diglycosylated PrP^F198S^ are acidic. Using Endo H, we confirmed that a large amount of glycans from PrP^T183A^ is immature, as originally reported by Capellari et al. [10], and our findings support the hypothesis that while only some PrP^T183A^ contains modified glycans (the ~50% Endo H resistant molecules), all of the PrP^T183A^ fails to reach the cell surface. The present study demonstrated that the glycans at either the first or second site were virtually completely digested by Endo H from monoglycosylated PrP^F198S^. Interestingly, the glycans of di-glycosylated PrP^F198S^ were not significantly digested, suggesting that di-glycosylated PrP^F198S^ are more mature than monoglycosylated forms.

Glycans in mouse and hamster brain PrP^Sc^ have been characterized by mass spectrometry in previous reports [26, 27]. Glycosylation at each of the two N-linked consensus sites of mouse ME7 strain was directly compared by mass spectrometry and differences in N-glycan populations between the two sites were observed [26]. Notably, although both sites contained major neutral components, many other minor neutral components were only observed at the first but not the second site. The majority of glycans at the first site were bi- and tri-antennary structures with a mass range of approximately 1.66-2.34 kDa, whereas the second site mainly contained tri- and tetra-antennary structures with a mass range of ~2.0-3.02 kDa [26]. Therefore, the molecular mass of glycans was greater in the second site than in the first site, having a difference ranging from 0.34-0.68 kDa. The range of oligosaccharides in PrP^C^ and PK-resistant PrP27-30 was found to be identical while the relative proportions of some glycans were different in the two species [27]. Compared to PrP^C^, PrP27-30 had a reduced bisected and an increased tri- and tetra-antennary glycans [27]. Nevertheless, to date no mass spectrometry analysis of the glycans on the human PrP^Sc^ or the PK-resistant human PrP has been reported, and the glycans on the two monoglycosylated species are not readily differentiated by Western blotting using regular anti-PrP antibodies such as 3F4.

V14 and V61 are well-characterized antibodies that were generated with the recombinant sheep PrP as the immunogen but are able to discriminate the two PrP species monoglycosylated either at the first or second glycosylation site of sheep, human, hamster and mouse [16]. In addition to recognizing the unglycosylated PrP, V14 also recognizes the PrP species monoglycosylated at the first site, while V61 recognizes the PrP species monoglycosylated at the second site. Using these two antibodies, it was confirmed that the molecular mass of glycans is greater at the second than the first site [16]. Although V14 also detected the di-glycosylated sheep PrP^C^ but it did not detect the di-glycosylated sheep PrP^Sc^. Our data indicated that the molecular mass of glycans is greater at the second than the first site on the human PK-resistant PrP^Sc^ from sCJD, similar to that observed in scrapie PrP^Sc^ [16]. However, the molecular mass of glycans at the second site of PrP^T183A^ seemed to be smaller than that of glycans on the second site of PrP^F198S^ or brain PrP^Sc^ of sCJD. This provides further evidence that the T183A mutation perturbs the glycosylation at the second site. Furthermore, our present study demonstrated that the PrP^F198S^ species monoglycosylated at the first and second sites were undetectable after the Endo H treatment. Therefore, the F198S mutation similarly alters glycosylation not only at the second, but also the first site. However, the glycans on the di-glycosylated PrP^F198S^ were not cleaved by Endo H, indicating that glycans on the di-glycosylated PrP are mature. Although the epitopes of V14, localized at sheep PrP188-199 corresponding to human PrP185-196, and V61, localized at sheep PrP169-174 corresponding to human PrP166-171, were mapped by studying the 2.5-Å resolution PrP-Fab crystal complex, the conventional peptide scanning failed to localize them on the PrP sequence [16, 18, 17]. As a result, the epitopes have been believed to be conformation-dependent [16], which is in agreement with our results reported here. We have demonstrated previously that reduction and alkylation blocks the accessibility of the 6H4 epitope but not epitopes of 3F4 and anti-C by breaking the disulfide bond of PrP [13]. In the current study, data revealed that the reduction of PrP by TBP decreased the accessibility of the V14 epitope and a combination of reduction and alkylation completely blocked its accessibility, providing further evidence that the accessibility of the V14 epitope depends on an intact disulfide bond on the C-terminal PrP. The V14 epitope was blocked by the F198S mutation as well, suggesting that the F198S mutation may also rupture the disulfide bond. These results parallel discoveries with other conformational sensitive anti-PrP antibodies, including 8H4, which was also reported to have the lower affinity for PrP^F198S^ than for PrP^Wt^ [11]. Unexpectedly, V61 detected no glycosylated and unglycosylated PrP species from all three types of cultured cells even though it detected brain PrP^C^ and PrP^Sc^. Why the V61 epitope becomes inaccessible in all wild-type and mutant PrP species remains to be determined. Since the amount of PrP^Wt^ used seemed to be large, indicated by V14 (Fig. 4B), it is most unlikely that inability of V61 to detect PrP from cultured cells mainly resulted from its lower affinity for PrP.

Autophagy degrades organelles and unfolded proteins through the lysosomal pathway and has been considered a key adaptive response that can prevent death in stressed or diseased cells [28]. This natural process normally occurs at a basal level in cells and it can be induced by a variety of conditions [29]. Using electron microscopy, we observed that autophagy was slightly increased in cells expressing PrP^F198S^ compared to cells expressing PrP^Wt^. Surprisingly, autophagy was dramatically decreased in cells expressing human PrP^T183A^, which further highlights a possible paradoxical relationship between autophagy and aggregation, since the largest amounts of PrP aggregates were also observed in cells expressing the T183A mutant. It is worth noting that cytosolic PrP aggregates were recently observed to activate reticulon 3 (RTN3), which inhibited autophagy and impeded the clearance of cytosolic PrP aggregates *in vitro* [30]. Furthermore, it was revealed that RTN3 activation was mediated by the enhanced interaction between Bcl-2 and beclin 1 [30]. Since autophagy is believed to play either a pro-survival or a pro-death role, determining the molecular mechanisms underlying the differences in the induction of autophagy using our cell models would present an important avenue for developing therapeutic strategies for protein folding disorders.

The physiology and pathophysiology of iPrP^C^ currently remain unclear. While it has been proposed that iPrP^C^ is involved in prion and Alzheimer diseases and in the long-term memory storage according to our recent findings [31-34], new insights into the mechanisms underlying the spontaneous formation of PrP aggregates obtained with cell models would be significant in enhancing our understanding of not only prion diseases but also other diseases involving protein misfolding.

## MATERIALS AND METHODS

### Reagents and antibodies

Phenylmethylsulfonyl fluoride (PMSF), PK, N, N'-diisopropylcarbodiimide, 1-hydroxybenzotriazole, trifluoroacetic acid, piperidine, triisobutylsilane, and dichloromethane were purchased from Sigma Chemical Co. (St. Louis, MO). Peptide N-glycosidase F (PNGase F) was purchased from New England Biolabs (Beverly, MA) and used according to the manufacturer's protocol. Reagents included Amino-PEG cellulose membranes and Fmoc-amino acids were obtained from Intavis (San Marcos, CA). N-α-Fmoc-O-benzyl-L-phosphoserine and N-α-Fmoc-O-benzyl-L-phosphothreonine were purchased from AnaSpec (San Jose, CA). Reagents for enhanced chemiluminescence (ECL Plus) were obtained from Amersham Pharmacia Biotech, Inc. (Piscataway, NJ). Magnetic beads (Dynabeads M-280, tosylactivated) were from Dynal Co. (Oslo, Norway). The recombinant human PrP23-231 was kindly provided by Dr. Witold K. Surewicz from the Department of Physiology and Biophysics, Case Western Reserve University. The gene 5 protein was kindly provided by Drs Geoff Kneale and John McGeehan from Biophysics Laboratories, Institute of Biomedical and Biomolecular Sciences, University of Portsmouth, Portsmouth, United Kingdom. Anti-PrP antibodies, including mouse monoclonal antibody 3F4 against human PrP residues 106-110 [12], 6H4 against human PrP145-152, 1E4 against human PrP 97-105 [6] (Cell Sciences, Inc., Canton, MA), V14 against human PrP185-196 [16], V61 against approximately before human PrP168 to residue 181 [16], anti-C against PrP220-231 and anti-N against PrP30-40 [19] were also used.

### Cloning and production of cell lines

M-17 human neuroblastoma cells were transfected with the episomal vector CEP4β containing a prion coding sequence using the cationic lipid DOTAP (Roche Applied Science) as previously described [9, 10, 11]. Transfected cells were grown as bulk selected hygromycin-resistant cultures at 37°C in OPTI-MEM with 5% calf serum supplement, iron-enriched (GIBCO-BRL) and 500 μg/ml Hygromycin B (Calbiochem, La Jolla, CA). For each experiment, cells were removed from the flask with trypsin, and counted; the same number of cells from each cell culture was seeded onto Petri plates. They were incubated overnight to ~95% confluence in complete medium supplemented with serum and antibiotics.

### Brain tissues and preparation of brain homogenates

Consent to use autopsy material for research purposes was obtained for all samples. Autopsy was performed within 20 h after death. Biopsy brain tissues were immediately frozen in liquid nitrogen, then transferred to −80°C for future use. The 10% (w/v) brain homogenates were prepared in 9 volumes of lysis buffer (10 mM Tris, 100 mM NaCl, 0.5% Nonidet P-40, 0.5% deoxycholate, 10 mM EDTA, pH 7.4). As required, brain homogenates were centrifuged at 1000 *g* for 10 min at 4°C to collect the supernatant (S1). For PK-digestion, samples were incubated with designated amounts of PK at 37°C for 1 h and the reaction was terminated through the addition of PMSF at a final concentration of 3 mM and boiling in SDS sample buffer (3% SDS, 2 mM EDTA, 4% β-mercaptoethanol, 10% glycerol, 50 mM Tris, pH 6.8) for 10 minutes.

### Cell lysis

After removal of the media, cells were rinsed three times with PBS and lysed in 1.2 ml of lysis buffer on ice for 30 min. The cell lysates were centrifuged at 1000 *g* for 10 min at 4°C to remove nuclei and cellular debris. The supernatant was incubated with 5.5 ml of pre-chilled methanol at −80°C for 2 h and centrifuged at 14 000 *g* for 30 min at 4°C. The pellet was resuspended in 100 μl of lysis buffer.

### Epitope mapping by peptide membrane arrays

The general methods for preparing multiple overlapping peptides bound to cellulose membranes have been described in detail [35, 6, 12]. After blocking with 5% skim milk in TBS-T at 37°C for 2 h, the prion peptide membrane was probed with 3F4 at 1:10,000, 1E4 at 1:500, V14 at 1:100, or V61 at 1:10 in 1% skim milk for 2 h at 37°C. The membrane was washed with TBS-T, then incubated at 37°C with 1:4,000 HRP-conjugated sheep anti-mouse IgG for 1 h. After a final wash and developing with ECL Western blotting detection reagent (Amersham Pharmacia), the membrane was visualized by using Bio-Rad Fluorescent Imager. The control membrane was probed only with HRP-conjugated sheep anti-mouse IgG without primary antibody.

### Specific capture of abnormal PrP by g5p

The g5p molecule (100 μg) was conjugated to 7 x 10^8^ tosyl activated magnetic beads in 1 ml of PBS at 37°C for 20 h [36, 4]. The g5p-conjugated beads were incubated with 0.1 % BSA in PBS to block non-specific binding. The prepared g5p beads were stable for at least 3 months at 4°C. The specific capture of PrP^Sc^ by g5p was performed as described [36, 4] by incubating S1 fractions and g5p conjugated beads (10 μg g5p/6 x 10^7^ beads) in 1 ml of binding buffer (3% Tween-20, 3% NP-40 in PBS, pH 7.5). After incubation with constant rotation overnight at room temperature, the PrP-containing g5p beads were collected with an external magnetic force and all unbound molecules in the solution were removed. Following three rinses in the wash buffer (2% Tween-20 and 2% Nonidet P-40 in PBS, pH 7.5), the g5p beads were resuspended in SDS sample buffer (3% SDS, 2 mM EDTA, 10% glycerol, 50 mM Tris, pH 6.8) and heated at 95°C for 5 min to release bound proteins.

### Reduction and Alkylation of PrP

Recombinant PrP was boiled in equal volume of 2 X sample buffer (6% sodium dodecyl sulfate (SDS), 5% β-mercaptoethanol (β-ME), 4 mM EDTA, 20% glycerol, 125 mM Tris-HCl, pH 6.8) as described previously [13]. The samples were incubated with a 5-fold volume of pre-chilled methanol at −20 °C for 2 h, followed by a centrifugation at 16,000 g for 20 min at 4 °C. Pellets were resuspended in 20 μl of TBP buffer (5 mM TBP, 8 M urea, 2% CHAPS, 20 mM Tris-HCl, pH 8.0) for 1 h at room temperature and then incubated in the dark with alkylating antitumor drugs for designated time. The samples were incubated with pre-chilled methanol and centrifuged at 16,000 g for 20 min at 4 °C. Finally, the pellets were resuspended in 20 μl samples buffer and subjected to SDS-PAGE and immunoblotting with various anti-PrP antibodies.

### Velocity sedimentation in sucrose step gradients

Cell lysates were incubated with an equal volume of 2% Sarcosyl for 30 min on ice. The sample was loaded atop 10-60% step sucrose gradients and centrifuged at 200,000 x g in the SW55 rotor for 1 h at 4°C as described with minor modification [4]. After centrifugation, the contents of the centrifuge tubes were sequentially removed from the top to the bottom to collect 11 fractions. Aliquots of 11 fractions were subjected to immunoblot analysis described below.

### Electronic microscopy of cultured neuronal cells

Cells cultured on the ACLAR^©^ embedding film (Electron Microscopy Sciences, Hatfield, PA) were fixed with 2.5% glutaraldehyde in 0.1 M Millonigs’ phosphate buffer (pH f.4) for two hours at room temperature. After a rinse in 0.1 M phosphate buffer, the cells were postfixed in ferrocyanide-reduced osmium tetroxide (Karnovsky, 1971) for two hours, then rinsed in distilled water. The cells were soaked overnight in acidified 0.25% uranyl acetate. Another rinse was followed by dehydration in ascending concentrations of ethanol, passage through propylene oxide, and embedment in Poly/Bed 812 (Polysciences, Warrington, PA). The cells on the ACLAR^©^ film were cross-sectioned. Thin sections were stained with acidified methanolic uranyl acetate, followed by staining with Sato's triple lead stain as modified by Hanaichi et al. [37] and examined in a JEOL 1200EX electron microscope.

### Immunofluorescence microscopy

For immunofluorescence staining of PrP without PK comparison, transfected cells expressing PrP^Wt^, PrP^T183A^, or PrP^F198S^ were grown overnight on Poly-D-lysine coated coverslips. The cells were fixed in 4% paraformaldehyde for 15 minutes at room temperature. Blocking was performed with PBST (10% Goat Serum, 2% T-20, 1% Triton X-100), for 1 hr at room temperature before rinsing with PBS. The cells were then incubated with 3F4 (1:4,000) or 1E4 (1:50) at room temperature, rinsed with PBS, followed with FITC-conjugated goat anti-mouse IgG at 1:320 (Sigma, St. Louis, MO) for 1 hour at room temperature under darkness. After washing with PBS, the coverslips were mounted on slides using fluorescent mounting medium fluoromount with DAPI (Dako, Carpenteria, CA). For imunofluorescence staining with PK comparison, wild type cells were grown under identical conditions to those described above. They were also fixed in 4% paraformaldehyde for 15 minutes at room temperature. The blocking step included PK (10 μg/mL) for the PK positive dishes along with PBST. All later conditions were constant, with the exception of solely using monoclonal antibody 3F4 (1:4,000).

### Western blot analysis

Samples were resolved on 15% Tris-HCl Criterion pre-cast gels (Bio-Rad) for SDS polyacrylamide gel electrophoresis at 150 V for ~80 min. The proteins on the gels were transferred to Immobilon-P membrane (PVDF, Millipore) for 2 h at 70V. The membranes were incubated for 2 h at room temperature with either 3F4 (1:40,000), 1E4 (1:1,000), 6H4 (1:6,000), V14 (1:500), V61 (1:500), anti-N (1:6,000) or anti-C (1:10,000) as the primary antibody for probing the PrP molecule. Following incubation with HRP-conjugated sheep anti-mouse IgG at 1:5,000 for monoclonal antibodies or donkey-anti rabbit IgG at 1:6,000, the PrP bands or spots were visualized on Kodak film by the ECL Plus in accordance with the manufacturer's protocol.

## References

[1] Prusiner SB (1998). Prions. Proc Natl Acad Sci U S A.

[2] Aguzzi A, Polymenidou M (2004). Mammalian prion biology: one century of evolving concepts. Cell.

[3] Jarrett JT, Lansbury PT (1993). Seeding “one-dimensional crystallization” of amyloid: a pathogenic mechanism in Alzheimer's disease and scrapie?. Cell.

[4] Yuan J, Xiao X, McGeehan J, Dong Z, Cali I, Fujioka H, Kong Q, Kneale G, Gambetti P, Zou W Q (2006). Insoluble aggregates and protease-resistant conformers of prion protein in uninfected human brains. J Biol Chem.

[5] Kuczius T, Grassi J, Karch H, Groschup MH (2007). Binding of N- and C-terminal anti-prion protein antibodies generates distinct phenotypes of cellular prion proteins (PrPC) obtained from human, sheep, cattle and mouse. FEBS J.

[6] Yuan J, Dong Z, Guo JP, McGeehan J, Xiao X, Wang J, Cali I, McGeer PL, Cashman NR, Bessen R, Surewicz WK, Kneale G, Petersen RB, Gambetti P, Zou WQ (2008). Accessibility of a critical prion protein region involved in strain recognition and its implications for the early detection of prions. Cell Mol Life Sci.

[7] Hall D, Edskes H (2004). Silent prions lying in wait: a two-hit model of prion/amyloid formation and infection. J Mol Biol.

[8] Tompa P, Friedrich P (1998). Prion proteins as memory molecules: a hypothesis. Neuroscience.

[9] Petersen RB, Parchi P, Richardson SL, Urig CB, Gambetti P (1996). Effect of the D178N mutation and the codon 129 polymorphism on the metabolism of the prion protein. J Biol Chem.

[10] Capellari S, Zaidi SI, Long AC, Kwon EE, Petersen RB (2000). The Thr183Ala Mutation, Not the Loss of the First Glycosylation Site, Alters the Physical Properties of the Prion Protein. J Alzheimers Dis.

[11] Zaidi SI, Richardson SL, Capellari S, Song L, Smith MA, Ghetti B, Sy MS, Gambetti P, Petersen RB (2005). Characterization of the F198S prion protein mutation: enhanced glycosylation and defective refolding. J Alzheimers Dis.

[12] Zou WQ, Langeveld J, Xiao X, Chen S, McGeer PL, Yuan J, Payne MC, Kang HE, McGeehan J, Sy MS, Greenspan NS, Kaplan D, Wang GX, Parchi P, Hoover E, Kneale G, Telling G, Surewicz WK, Kong Q, Guo JP (2010). PrP conformational transitions alter species preference of a PrP-specific antibody. J Biol Chem.

[13] Yuan J, Kinter M, McGeehan J, Perry G, Kneale G, Gambetti P, Zou WQ (2005). Concealment of epitope by reduction and alkylation in prion protein. Biochem Biophys Res Commun.

[14] Shakin-Eshleman SH, Spitalnik SL, Kasturi L (1996). The amino acid at the X position of an Asn-X-Ser sequon is an important determinant of N-linked core-glycosylation efficiency. J Biol Chem.

[15] Tzaban S, Friedlander G, Schonberger O, Horonchik L, Yedidia Y, Shaked G, Gabizon R, Taraboulos A (2002). Protease-sensitive scrapie prion protein in aggregates of heterogeneous sizes. Biochemistry.

[16] Moudjou M, Treguer E, Rezaei H, Sabuncu E, Neuendorf E, Groschup MH, Grosclaude J, Laude H (2004). Glycan-controlled epitopes of prion protein include a major determinant of susceptibility to sheep scrapie. J Virol.

[17] Rezaei H, Eghiaian F, Perez J, Doublet B, Choiset Y, Haertle T, Grosclaude J (2005). Sequential generation of two structurally distinct ovine prion protein soluble oligomers displaying different biochemical reactivities. J Mol Biol.

[18] Eghiaian F, Grosclaude J, Lesceu S, Debey P, Doublet B, Tréguer E, Rezaei H, Knossow M (2004). Insight into the PrPC-->PrPSc conversion from the structures of antibody-bound ovine prion scrapie-susceptibility variants. Proc Natl Acad Sci U S A.

[19] Chen SG, Teplow DB, Parchi P, Teller JK, Gambetti P, Autilio-Gambetti L (1995). Truncated forms of the human prion protein in normal brain and in prion diseases. J Biol Chem.

[20] Zhou X, Bi H, Wong J, Shimoji M, Yuan J, Xiao X, Zou WQ (2011). Alkylating antitumor drug mechlorethamine conceals structured PrP domain and inhibits in vitro prion amplification. J Toxicol Environ Health.

[21] Puckett C, Concannon P, Casey C, Hood L (1991). Genomic structure of the human prion protein gene. Am J Hum Genet.

[22] Nitrini R, Rosemberg S, Passos-Bueno MR, da Silva LS, Iughetti P, Papadopoulos M, Carrilho PM, Caramelli P, Albrecht S, Zatz M, LeBlanc A (1997). Familial spongiform encephalopathy associated with a novel prion protein gene mutation. Ann Neurol.

[23] Tagliavini F, Prelli F, Ghiso J, Bugiani O, Serban D, Prusiner SB, Farlow MR, Ghetti B, Frangione B (1991). Amyloid protein of Gerstmann-Sträussler-Scheinker disease (Indiana kindred) is an 11 kd fragment of prion protein with an N-terminal glycine at codon 58. EMBO J.

[24] Rogers M, Taraboulos A, Scott M, Groth D, Prusiner SB (1990). Intracellular accumulation of the cellular prion protein after mutagenesis of its Asn-linked glycosylation sites. Glycobiology.

[25] Lehmann S, Harris DA (1997). Blockade of glycosylation promotes acquisition of scrapie-like properties by the prion protein in cultured cells. J Biol Chem.

[26] Stimson E, Hope J, Chong A, Burlingame AL (1999). Site-specific characterization of the N-linked glycans of murine prion protein by high-performance liquid chromatography/electrospray mass spectrometry and exoglycosidase digestions. Biochemistry.

[27] Rudd PM, Endo T, Colominas C, Groth D, Wheeler SF, Harvey DJ, Wormald MR, Serban H, Prusiner SB, Kobata A, Dwek RA (1999). Glycosylation differences between the normal and pathogenic prion protein isoforms. Proc Natl Acad Sci U S A.

[28] Nixon RA (2006). Autophagy in neurodegenerative disease: friend, foe or turncoat?. Trends Neurosci.

[29] Mizushima N, Klionsky DJ (2007). Protein turnover via autophagy: implications for metabolism. Annu Rev Nutr.

[30] Chen R, Jin R, Wu L, Ye X, Yang Y, Luo K, Wang W, Wu D, Ye X, Huang L, Huang T, Xiao G (2011). Reticulon 3 attenuates the clearance of cytosolic prion aggregates via inhibiting autophagy. Autophagy.

[31] Zou WQ, Puoti G, Xiao X, Yuan J, Qing L, Cali I, Shimoji M, Langeveld JP, Castellani R, Notari S, Crain B, Schmidt RE, Geschwind M, Dearmond SJ, Cairns NJ, Dickson D, Honig L, Torres JM, Mastrianni J, Capellari S, Giaccone G, Belay ED, Schonberger LB, Cohen M, Perry G, Kong Q, Parchi P, Tagliavini F, Gambetti P (2010). Variably protease-sensitive prionopathy: a new sporadic disease of the prion protein. Ann Neurol.

[32] Zou WQ (2010). Chameleon-like prion protein and human cognition. Curr Topics in Biochem Res.

[33] Zou WQ, Xiao X, Yuan J, Puoti G, Fujioka H, Wang X, Richardson S, Zhou X, Zou R, Li S, Zhu X, McGeer PL, McGeehan J, Kneale G, Rincon-Limas DE, Fernandez-Funez P, Lee HG, Smith MA, Petersen RB, Guo JP (2011). Amyloid-{beta}42 Interacts Mainly with Insoluble Prion Protein in the Alzheimer Brain. J Biol Chem.

[34] Zou WQ, Zhou X, Yuan J, Xiao X (2011). Insoluble cellular prion protein and its association with prion and Alzheimer diseases. Prion.

[35] Guo JP, Petric M, Campbell W, McGeer PL (2004). SARS corona virus peptides recognized by antibodies in the sera of convalescent cases. Virology.

[36] Zou WQ, Cashman NR (2002). Acidic pH and detergents enhance in vitro conversion of human brain PrPC to a PrPSc-like form. J Biol Chem.

[37] Hanaichi T, Sato T, Iwamoto T, Malavasi-Yamashiro J, Hoshino M, Mizuno N (1986). A stable lead by modification of Sato's method. J Electron Microsc (Tokyo).

